# Effects of Rapid Palatal Expansion on Chewing Biomechanics in Children with Malocclusion: A Surface Electromyography Study

**DOI:** 10.3390/s20072086

**Published:** 2020-04-07

**Authors:** Fabiola Spolaor, Martina Mason, Alberto De Stefani, Giovanni Bruno, Ottavia Surace, Annamaria Guiotto, Antonio Gracco, Zimi Sawacha

**Affiliations:** 1 Department of Information Engineering, University of Padova, Via Gradenigo, 6-35121 Padua, Italy; ottavia.surace@gmail.com (O.S.); guiotto@dei.unipd.it (A.G.); zimi.sawacha@unipd.it (Z.S.); 2 Department of Neuroscience, School of Dentistry, University of Padua, Via Giustiniani, 2-35128 Padua, Italy; martina.mason@alice.it (M.M.); alberto.de.stefani@hotmail.it (A.D.S.); giobruno93@gmail.com (G.B.); Antonio.gracco@unipd.it (A.G.); 3 Department of Medicine, University of Padova, Via Giustiniani, 2-35128 Padova, Italy

**Keywords:** posterior crossbite, children, electromyography, rapid palatal expansion

## Abstract

Malocclusion during childhood may affect both morphology and masticatory function and could greatly affect the subsequent growth and development of the jaws and face. The purpose of this study was to evaluate the efficiency of surface electromyography in describing the effects of the rapid palatal expansion (RPE) on Masseter (M) and Temporalis Anterior (T) muscles’ activity in 53 children with different types of malocclusion: bilateral posterior crossbite (BPcb), underdeveloped maxillary complex without crossbite (NOcb) and unilateral posterior crossbite on the right (UPCBr) and on the left (UPCBl). The muscular activities during chewing tasks were assessed bilaterally before and after RPE application and three months after removal. Both the envelope’s peak (µV) and its occurrence (% of chewing task) were extracted from the surface electromyography signal. Our results showed the presence of statistically significant differences (p < 0.05) on temporomandibular joint muscles, across different assessments, in all the tested populations of subjects. Surface electromyography demonstrated a relationship between the correction of a maxillary transverse discrepancy and the restoration of a muscle’s activation patterns comparable to healthy subjects for both T and M.

## 1. Introduction

Understanding the characteristic features of primary dentition, as well as the changes that take place in the transitional stage from primary to permanent dentition for a particular population, is essential for dentists involved in planning early preventive and interceptive orthodontic treatment [1,2,3,4]. Posterior crossbite is a frequent malocclusion associated with maxillary transverse discrepancy (MTD), in the early mixed dentition stage [5,6,7,8] that normally persists from childhood to adulthood if not treated [9]. In most cases, it is accompanied by a mandibular lateral shift during closure [10] that can determine an abnormal mandibular growth [11,12], asymmetrical jaw muscle activity [13,14,15,16], changes in mandibular movements [17] and temporomandibular disorders (TMD) [18,19]. Children with unilateral posterior crossbite exhibit different types of unusual chewing patterns [20], the most frequent of which is the reverse sequence cycle [21]. Rapid palatal expansion (RPE) is an evidence-based procedure that has been demonstrated to determine an orthopedic effect on the maxillary complex [3,4]. It can induce equilibrium in the masticatory muscles and consequent further harmonious development of the jaws, working on the relationship between functional and morphological aspects of the malocclusion. [15,22]. 

Surface electromyography (sEMG) analysis provides important information on muscular conditions, both in dynamic and static contraction. In the context of MTD, several authors have analyzed sEMG’s characteristics during different dynamic tasks both in subjects with and without malocclusion. SEMG is considered the most objective and reliable diagnostic tool for assessing changes in the electrical activity of the masticatory muscles [23,24,25,26,27,28,29,30,31]. Several authors focused their research on the application of sEMG in adults with different types of TMD to analyze the modification of muscular activity during clenching and chewing with specific occlusal prostheses and to standardize the sEMG recordings in healthy subjects [32,33]. In 2011 Tecco et al. [22] found some associations between the cephalometric variables and the sEMG activity of the head, neck and trunk muscles [22]. In 2008 Dong et al. [34] tested the hypothesis that developmental mandibular asymmetry is associated with increased asymmetry in muscle activity in adult patients [34]. This asymmetric activation was interpreted as a compensatory strategy to achieve stability during masticatory function and some sEMG indices of asymmetry were proposed [8,15]. Nevertheless, attention has been paid to the analysis of sEMG in patients with different types of TMD or orofacial pain [35,36]. The majority of these studies focused their attention on adult subjects, healthy or with TMD, while only a few studies focused on the application of sEMG in children. In this case, authors considered alterations other than crossbite such as sleep bruxism [17] and interceptive orthodontics on orbicular muscle activity [37,38]. Finally, even though monitoring muscular activity during a functional treatment has been recognized as a useful tool in guiding the therapy [24,25,28,29,30,31,32,33], its clinical usefulness still represents a critical factor in its general acceptance. RPE represents the gold standard treatment for children with MTD in early mixed dentition but its effects on the muscular system are still controversial [14,34,35,36,37,38,39,40].

Our aim is twofold: firstly, to provide a methodology to verify the ability of RPE to promote selective activity of the temporomandibular muscles and for assessing the possible compensatory mechanisms adopted. Secondly, to highlight differences in sEMG activity as a response to RPE application across three populations of children with different malocclusions: bilateral posterior crossbite (BPcb), underdeveloped maxillary complex without crossbite (NOcb), unilateral posterior crossbite on the right (UPCBr) and on the left (UPCBl), and a control group of healthy subjects (C).

## 2. Material and Methods 

### 2.1. Subjects

**Figure 1:**
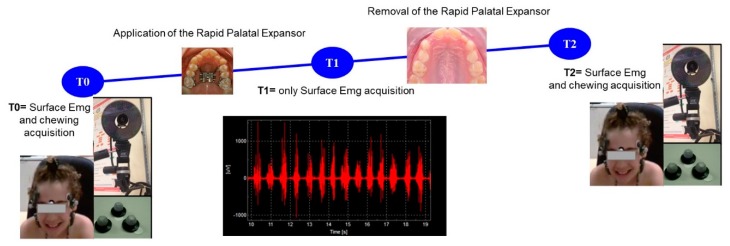
Timeline of the project.

53 children (26 girls and 27 boys) were enrolled in the study (the protocol was approved by the Ethical Committee of the University Dental Clinic of Padova, Protocol n° 3493/AO/15). They were divided into two groups: 43 patients with different types of malocclusion and 10 healthy controls (C) (Figure 1). The first group was further divided into 3 subgroups: 10 BPcb (mean age 9 ± 1.51; mean BMI 18.97 ± 2.98); 15 UPcb children (9 UPCBr and 6 UPCBl, mean age 9 ± 2.28; mean BMI 17.64 ± 3.95); 18 NOcb children (mean age 10 ± 1.72; mean BMI 17.36 ± 2.33). The sample size was defined through the Altman normogram, accordingly to Manfredini et al. [41], by taking into account several parameters other than sEMG, as this work was part of a wider project that involved several variables such as sEMG, gait kinematics, space and time parameters, and plantar pressure analysis. Clinical characteristics were reported in Table 1.

### 2.2. Instrumental Protocol

Acquisition sessions took place at the BioMov - Lab (Department of Information Engineering of the University of Padua) using the following instrumentation:

- 6-camera motion capture system 60–120 Hz (BTS Bioengineering, Quincy, MA US)
- An 8-channel Free EMG system 1000 Hz (BTS Bioengineering, Quincy, MA, US)
- Two web cameras (30 Hz, Microsoft Corporation, Redmond, WA, US)

All instruments were synchronized. The sEMG activity was collected bilaterally on the following muscles: Masseter ((M), right (RM) and left (LM)), Temporalis Anterior ((T) right (RT) and left (LT)), 10-mm diameter pre-gelled sEMG sensors were positioned on the muscles in accordance with Castroflorio et al. [2]. 

### 2.3. Acquisition Protocol

After signing an Informed Consent form, the following treatment and acquisitions protocol took place (Figure 1):

1. **T0**. Before the start of the treatment every subject underwent the sEMG analysis
2. Start of treatment with RPE according to individual clinical condition
3. **T1**. Stopping of the expansion (between 4 and 6 weeks after treatment start)
4. Removal of RPE 6 months after T1.
5. **T2**. Three months after the RPE removal we performed a check-up visit to evaluate changes and the sEMG analysis was repeated to identify any further change in masticatory strategy.

During sEMG acquisition at T0, T1 and T2, children were sitting on a chair and were asked to chew the same kind of biscuit. The registrations of muscle activity lasted the time necessary to swallow the biscuit. 

### 2.4. Data Analysis

The sEMG recorded signals were bandpass filtered between 10 and 450 Hz with a 5th order Butterworth filter and wave rectified. The envelope was computed (Matlab R2013a) by low-pass filtering the signals with 4th order Butterworth filter and a cut-off frequency of 5 Hz as in (30), hence the Peak of the Envelope (PoE in µV) was normalized on the mean value of the total sEMG signal, meanwhile, the Position of the Peak of the Envelope (PPoE) was calculated as a percentage of the chewing (0–100%). 

Ten chewing cycles were extracted from each trial through a Matlab custom code (v. R 2013a) by considering markers trajectories and video sequences.

### 2.5. Statistical Analysis

One-way ANOVA or Kruskal Wallis tests were performed after evidencing a normal distribution (Levene’s Test) using SPSS (v. 19.0) and sEMG activity was compared across all subject groups. Paired sample T tests were performed to compare results of the different acquisition’s sessions within each group of subjects. Comparisons were made before RPE application, after RPE application and after the removal. A significance level of p < 0.05 was adopted for all statistical analysis, using a Bonferroni correction when appropriate

## 3. Results

**Figure 2:**
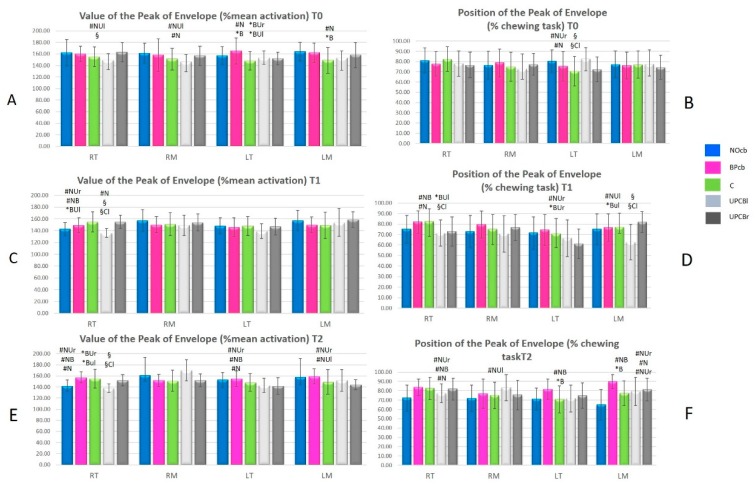
the results of one-way ANOVA (p < 0.05) at T0 (**A**,**B**), at T1 (**C**,**D**), at T2 (**E**,**F**) between the 5 populations. Color Legend: blue for NoCB, pink for BPcb, green for Controls, light grey for UPCBl and grey for UPCBr.

**Figure 3:**
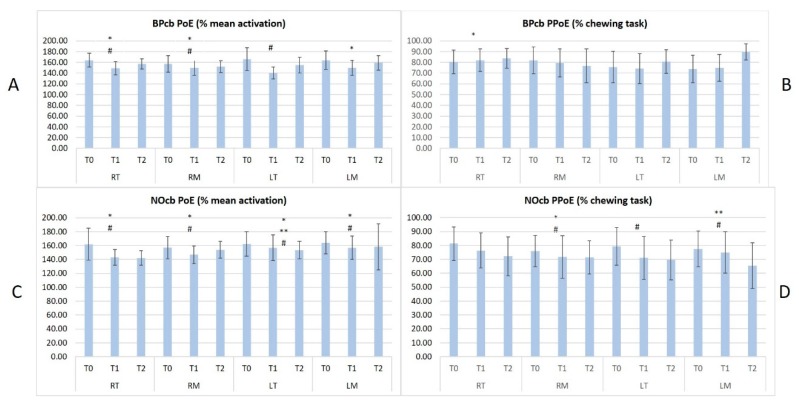
the results of a paired T Test (p < 0.05) at T0, T1 and T2 in both BPcb in the upper graphs (**A**,**B**), and in NOcb (in the lower graphs (**C**,**D**)).

**Figure 4:**
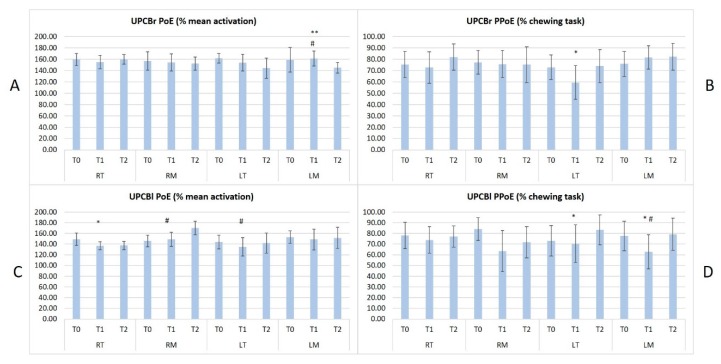
the results of a paired T Test (p < 0.05) at T0, T1 and T2 in both UPCBl in the upper graphs (**A**,**B**), and in UPCBr in the lower graphs (**C**,**D**).

All patients concluded the study. No significant differences were observed in term of participants’ age, BMI and sex across groups. All results in terms of PoE and PPoE between the different groups are collected in Figure 2, Figure 3 and Figure 4.

In Figure 2A,C,E the y-axis represents the Peak of Envelope (PoE in % of mean activation) in terms of mean and standard deviation; in Figure 2B,D,F the y-axis represents the Position of Peak of Envelope (PPoE % of chewing task) in terms of mean and standard deviation.

On the x-axis of all graphs the names of the muscles are reported: RT (Right Temporalis), RM (Right Masseter), LT (Left Temporalis) and LM (Left Masseter).

The significant results of one-way ANOVA (p < 0.05) between groups are shown with different symbols as in Table 2.

In Figure 3A,C the y-axis represents the Peak of Envelope (PoE in % of mean activation) in terms of mean and standard deviation; in Figure 3B,D the y-axis represents the Position of Peak of Envelope (PPoE % of chewing task) in terms of mean and standard deviation.

On the x-axis of all the graphs, the names of the muscles are reported: RT (Right Temporalis), RM (Right Masseter), LT (Left Temporalis) and LM (Left Masseter) and for each muscle, the acquisition time is reported (T0, T1 and T2).

The significant results of paired T Test (p < 0.05) between different acquisitions (T0, T1 and T2) are shown with different symbols as follows: T0 vs. T1, p < 0.05 = *; T1 vs. T2, p < 0.05 = **; T0 vs. T2, p < 0.05= #.

In Figure 4A,C the y-axis represents the Peak of Envelope (PoE in % of mean activation) in terms of mean and standard deviation; in Figure 4B,D the y-axis represents the Position of Peak of Envelope (PPoE % of chewing task) in terms of mean and standard deviation.

On the x-axis of all graphs the names of the muscles are reported: RT (Right Temporalis), RM (Right Masseter), LT (Left Temporalis) and LM (Left Masseter) and for each muscle the acquisition time is reported (T0, T1 and T2). The significant results of Paired T-Test (p < 0.05) between different acquisitions (T0, T1 and T2) are shown with different symbols as follows: T0 vs. T1, p < 0.05 = *; T1 vs. T2, p < 0.05 = **; T0 vs. T2, p < 0.05 = #.

T0 measurements highlighted significant differences in the muscular activities in terms of mean activation and occurrence within the chewing tasks across all the malocclusions and in comparison with the control group. These results demonstrated how the skeletal framework can influence masticatory function in children. 

In particular, symmetrical malocclusions (BPcb and NOcb) presented symmetrical and similar muscular function while the patients affected by unilateral crossbite presented a lower activity in terms of PoE and earlier activation in terms of PPoE on the contralateral side. Furthermore, BPcb and NOcb presented a slightly increased activity when compared to the control group. 

At T1 (4/6 weeks) sEMG showed the highest variability in comparison with T0 and T2. The authors expected this result since the muscular activity generally needs a longer time to adapt to the new skeletal framework. Furthermore, other parameters can influence the muscular activity at this time such as altered or earlier occlusal contacts, dental and skeletal pain due to the active maxillary expansion and the presence of the appliance bonded in the mouth. 

At T2 (3 months after the RPE removal) some differences in sEMG parameters emerged between the different malocclusions. In terms of PoE, all the muscles reduced their mean activity except for the RM in UPCBl patients. The authors expected a similar or lower activity at T2 in all the malocclusions due to the improvement determined by the orthodontic treatment. 

In terms of PPoE, NOcb had the best improvement showing a significant reduction in RM, LT, and LM and a non-significant reduction in RT. The chewing task does not significantly change in the other malocclusions. The authors expected this result for two main reasons. First of all, NOcb is the easiest malocclusion to correct and generally requires fewer RPE activations. Secondly, the correction often determines earlier contacts on first molars due to the transversal dental compensation present at T0.

## 4. Discussion

The posterior crossbite is one of the most frequent malocclusions in children [4] and does not present a tendency towards spontaneous correction. For this reason, it should be treated with maxillary expansion as early as possible, after an accurate orthodontic diagnosis. The RPE promotes skeletal and dental positive effects, such as alignment, leveling, and intercuspation. It often corrects the anterior crossbite as noted by Haas, 1961 [42]. 

The results of our study highlighted two main points: first of all, sEMG is an efficient tool to evaluate and compare masticatory muscle strategies in children affected by maxillary transversal discrepancy. Furthermore, we have demonstrated a positive effect of RPE treatment on the muscular activity in all the malocclusions, particularly in BPcb and NOcb. Other improvements in these populations have been demonstrated in our previous study [43]. Indeed, the higher muscle activation observed at T0 between BPcb/NOcb and the healthy subjects was no longer observed after RPE removal. This initial alteration could be interpreted as the need for recruiting more muscle fibers to accomplish the same task as a consequence of maxillary transverse discrepancy. The reduction in sEMG activity, after the RPE application, supports the hypothesis that the procedure reestablishes a more-functional masticatory activity.

Alterations in sEMG activity were already recorded by Troelstrup et al, 1970 [44] in the group of UPCB subjects on the posterior temporal muscles of the side with the crossbite. However, in the same subjects, no differences were observed on the activity of the masseter [38]. Results of our study for the first time not only compared the activity of masticatory muscles on BPcb and NOcb subjects with healthy subjects, but also recorded a significantly higher activation of both T and M. Furthermore, besides assessing muscle impairment, sEMG showed the positive effect of RPE in restoring a more physiological pattern of these muscles. In 2010 Andrade et al. [45] analyzed a group of UPcb children and observed an altered coordination of the masticatory muscles while chewing. In 2015 Woźniak et al. [8,31] evaluated the tone of both the M and T muscles in growing children before and after six months of treatment with a removable orthodontic appliance, and their results indicated that the electrical activity of the muscles was the same in the group of girls and boys. Furthermore, they observed that in the mandibular rest position and maximum voluntary contraction, the activity of the T was significantly higher than the M. 

Despite the good results concerning the use of the RPE in children with different types of occlusions, our study presents some limits. First of all, the small number of patients and their distribution in the four groups, which were not homogeneous. Secondly, the relationship between chewing tasks and maximum voluntary contraction will be the subject of future developments. 

There is a wide literature on the clinical applications of sEMG and its relevance to evaluate the activity of masticatory muscles [24,25,27,38]. Previous works highlighted the diagnostic usage of sEMG in patients with UPCB [13,35,38,46], tooth grinding [47], developmental mandibular asymmetry [35,38], sleep bruxism [17], reverse and non-reverse chewing, class II malocclusion [48] and open bite malocclusion [11,26]. Al-Saleh et al. proposed sEMG as a tool for diagnosing TMD [49], meanwhile, other authors used sEMG as an aid to differentiate them from neck disorders [50,51,52]. Woźniak et al., in their study proposed sEMG as a tool to evaluate the impact of functional appliances on muscle activity in patients with TMD and UPCB [31]. 

To the authors’ knowledge, our study analyzed for the first time the sEMG of masticatory muscles in BPcb, UPCB and NOcb patients with a control group of healthy children.

Besides the observation of sEMG activity in these subjects, while performing a functional task such as chewing, our study highlighted the positive impact of the orthodontic treatment with RPE in correcting the observed sEMG alterations. For this reason, the positive effects of the RPE treatment are not only on the dentition, skeletal framework, and nasal airways but also on the functional activities of the masticatory muscles. Hence sEMG could be used to assess improvements of the masticatory muscles and for personalizing RPE dental treatment plans. 

In conclusion, when considering the improvement both in the timing (PPoE) and the amount of activity (PoE) registered in each group of subjects, a relationship existed between the correction of a maxillary transverse discrepancy and the restoration of a muscle’s activation patterns comparable to healthy subjects for both T and M. In agreement with Nishi et al. [15] sEMG represents a useful tool in general dentistry not only for observation purposes, but also for diagnosis and measurements of the treatment outcomes.

## Figures and Tables

**Table 1:** clinical and anamnestic data of our population.

	BPCB		NoCB		UPCBr		UPCBl		C	
	Mean	St.Dev	Mean	St.Dev	Mean	St.Dev	Mean	St.Dev	Mean	St.Dev
**N°**	10		18		9		6		10	
**Age**	9.40	1.51	10.61	1.72	9.67	2.55	8.25	2.60	9.80	2.20
**Height (m)**	1.30	0.10	1.32	0.34	1.27	0.51	1.07	0.49	1.36	0.09
**Weight (kg)**	31.17	7.60	33.44	6.71	38.89	18.89	32.31	10.24	34.00	5.14
**BMI (kg/m^2^)**	18.97	2.98	17.36	2.33	17.45	4.52	16.00	5.79	18.44	1.38

**Table 2:** symbols legend of one-way ANOVA significant results.

Comparison	Symbols in Figure 3
NOcb vs. BPcb	#NB
Nocb vs. C	#N
Nocb vs. UPCBr	#Nur
Nocb vs. UPCBl	#Nul
BPCB vs. C	*B
BPCB vs. UPCBr	*Bur
BPCB vs. UPCBl	*Bul
UPCBr vs. UPCBl	§
UPCBr vs. C	§Cr
UPCBl vs. C	§Cl
